# Can the sustainable development goals for cancer be met in Brazil? A population-based study

**DOI:** 10.3389/fonc.2022.1060608

**Published:** 2023-01-10

**Authors:** Marianna De Camargo Cancela, Dyego Leandro Bezerra de Souza, Luís Felipe Leite Martins, Leonardo Borges, Arthur Orlando Schilithz, Paul Hanly, Linda Sharp, Alison Pearce, Isabelle Soejomataram

**Affiliations:** ^1^ Division of Surveillance and Data Analysis, Coordination of Prevention and Surveillance, Brazilian National Cancer Institute, Ministry of Health, Rio de Janeiro, Brazil; ^2^ Department of Collective Health, Universidade Federal do Rio Grande do Norte, Natal, Brazil; ^3^ School of Business, National College of Ireland, Dublin, Ireland; ^4^ Population Health Sciences Institute, Newcastle University Centre for Cancer, University of Newcastle, Newcastle Upon Tyne, United Kingdom; ^5^ Daffodil Centre, The University of Sydney, a joint venture with Cancer Council NSW, School of Public Health, Faculty of Medicine and Health, The University of Sydney, Sydney, NSW, Australia; ^6^ Cancer Surveillance Unit, International Agency for Research on Cancer, Lyon, France

**Keywords:** sustainable development goals, cancer premature mortality, mortality predictions, mortality trends, cancer epidemiology, population-based

## Abstract

**Background:**

A one-third reduction in premature mortality (30-69 years) from chronic noncommunicable diseases is goal 3.4 of the United Nations Sustainable Development Goals (UN SDG). The burden of NCDs is expected to continue to increase in low- and middle-income countries, including Brazil.

**Objectives:**

The aim of this study was to assess geographical and temporal patterns in premature cancer mortality in Brazil between 2001 and 2015 and to predict this to 2030 in order to benchmark against the 3.4 SDG target.

**Methods:**

We used data on deaths from cancer in those aged 30-69, by age group, sex and cancer site, between 2001 and 2015 from the National Mortality Information System of Brazil (SIM). After correcting for ill-defined causes, crude and world age-standardised mortality rates per 100,000 inhabitants were calculated nationally and for the 5 regions. Predictions were calculated using NordPred, up to 2030.

**Results:**

The difference in observed (2011-2015) and predicted (2026-2030) mortality was compared against the SDG 3.4 target. Between 2011-2015 and 2026-2030 a 12.0% reduction in premature cancer age-standardised mortality rate among males and 4.6% reduction among females is predicted nationally. Across regions this varied from 2.8% among females in North region to 14.7% among males in South region. Lung cancer mortality rates are predicted to decrease among males but not among females nationally (men 28%, females 1.1% increase) and in all regions. Cervical cancer mortality rates are projected to remain very high in the North. Colorectal cancer mortality rates will increase for both sexes in all regions except the Southeast.

**Conclusions and recommendation:**

Cancer premature mortality is expected to decrease in Brazil, but the extent of the decrease will be far from the SDG 3.4 target. Nationally, only male lung cancer will be close to reaching the SDG 3.4 target, reflecting the government’s long-term efforts to reduce tobacco consumption. Projected colorectal cancer mortality increases likely reflect the epidemiological transition. This and, cervical cancer control will continue to be major challenges. These results will help inform strategic planning for cancer primary prevention, early detection and treatment programs; such initiatives should take cognizance of the regional differences highlighted here.

## Introduction

Non-communicable Diseases (NCD) were responsible for 15 million premature deaths (30-69 years) worldwide in 2016, with more than 85% of these deaths occurring in low- and middle-income countries (1). Cancer is responsible for 9.0 million deaths annually, second only to cardiovascular disease (CVD) (17.9 million deaths annually) as the leading cause of NCD death globally (2). The burden of NCDs is expected to continue to increase in low- and middle-income countries. The estimated economic losses associated with premature deaths - many of which are due to NCDs - are expected to rise to about US$ 7 trillion in these countries over the next 15 years (3).

Population ageing has accelerated internationally and most NCDs are more prevalent among older people. In addition, environmental and lifestyle factors increase the risk of death from NCDs (4), and important trends in these (such as the increase in obesity observed in many countries) have significant implications for the future burden of NCDs. As part of the strategy to curb the growing burden of NCDs, the United Nations (UN) has set, as part of the Sustainable Development Goals (SDGs), a global target to reduce total premature mortality from NCDs by one-third by 2030 (5). While actions to curb CVDs deaths have been successful in many world regions, cancer control has been challenging due to heterogeneity in the aetiology, natural history and pathology of the many different diseases that comprise cancer; cancer therefore deserves special attention with respect to the SDGs (6).

Brazil is a country with continental dimensions and has a population of approximately 210 million. It has the largest universal public health system in the world which, because of its size, faces difficulties in the structure and provision of services. Private health insurance is available in parallel and used by those citizens who can afford it - approximately 24% of the population, in 2019 (7). The historical and political background contributed to disparities in development across the regions of Brazil. The North and Northeast regions have lower social and health indicators, compared to the more economically and socially developed Southeast, South and Midwest regions. These differences affect the structure of care and access to health services (8, 9).

While general NCD-related mortality in Brazil is declining, and the country is likely to achieve the target of a one-third reduction by 2030 (1), the more complex situation with respect to regional disparity in reductions of the cancer burden remains unknown both nationally and regionally.

Examination of actual and projected numbers of cancers cases and deaths is essential in order to effectively plan, monitor and evaluate implementation of cancer control strategies. Given the important regional differences in health status, socioeconomic factors and health care access, detailed projections are needed at both the national and regional level in Brazil to ensure equitable design and implementation of cancer strategies and hence alignment with the key guiding principle of the sustainable development agenda.

The aim of this study was to assess geographical and temporal patterns in premature cancer mortality in Brazil between 2001 and 2015 and to predict this to 2030 in order to benchmark against the SDG 3.4 target i.e. one-third reduction in premature mortality.

## Methods

### Study design and data sources

**Figure 1:**
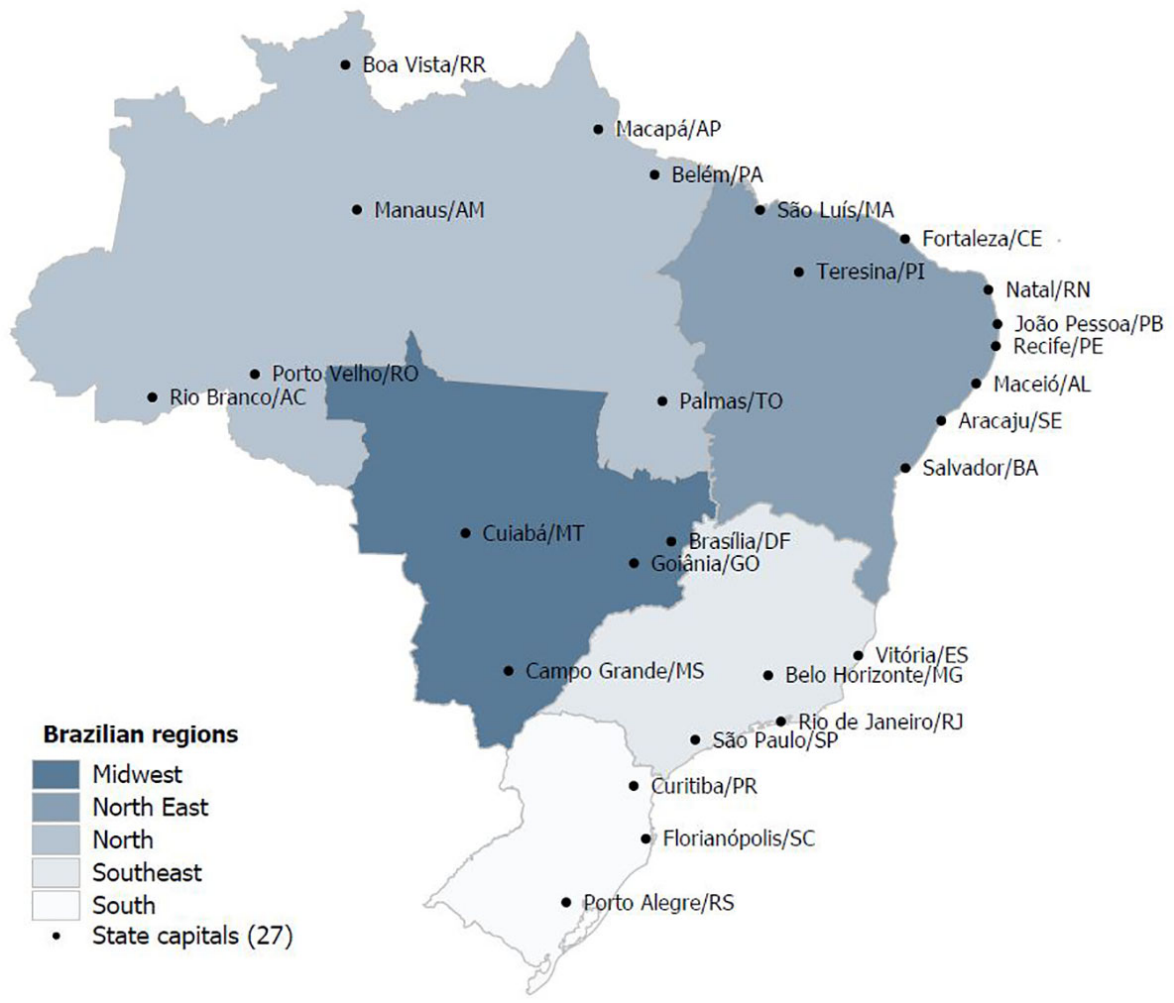
Brazilian geographical regions.

We performed an observational temporal series study based on secondary data from the Mortality Information System (SIM) of the Department of Informatics of the Brazilian Public Health System, which is publicly available for download (10). Brazil is a Federative Republic composed of 26 states and one federal district which are grouped in five geographical regions: North, Northeast, South, Southeast and Midwest, with a high human development index in 2020 (0.765) (11). The country has a population of 212 million (2020) according to the Brazilian Institute of Geography and Statistics (IBGE). The total Gross Domestic product (GDP) was approximately 1.4 trillion in 2020 with a relatively low level of income equality revealed by the Gini coefficient of 0.52. The geographical regions and their characteristics are shown in **Figure 1** and **Table 1**.

**Table 1:** Sociodemographic characteristics, Brazil and geographical regions.

	Brazil	North	Northeast	Southeast	South	Midwest
Area (Km^2^)	8,515,767	3,853,677	1,544,292	924,620	576,775	1,606,404
Number of States*	26	7	9	4	3	3
Total population	212,077,375	18,583,035	58,174,912	88,601,482	30,221,606	16,496,340
Population density (per Km^2^)	24.90	4.82	37.67	95.82	52.40	10.27
Human Development Index (2010)	0.765	0.667	0.663	0.766	0.754	0.757
Gini Index (2020)	0.524	0.543	0.526	0.517	0.457	0.496
Gross domestic product 2017 (%)	100.0%	5.6%	14.5%	52.9%	17.0%	10.0%
*Except Federal District						

Source IBGE (11).*Except Federal District.

The coverage of death registration in Brazil is between 90-99% (12). We selected all cancer deaths (except non-melanoma skin cancer) among those aged 30-69 between 2001 and 2015. The specific underlying cause of death was coded according to the International Classification of Diseases, 10th Revision (ICD-10). Ill-defined causes of death were redistributed as recommended (13) as were unspecified codes for deaths from uterine and digestive tract cancers (14, 15). Population data by region and age-group were obtained from demographic census and inter-census projections, from the Brazilian Institute of Geography and Statistics (11).

### Data analysis

The analyses were stratified by sex, selected topography – mouth and pharynx (C00-C14), oesophagus (C15), stomach (C16), colorectum (C18-21), liver (C22), gallbladder (C23-24), pancreas (C25), larynx (C32), lung (C33-34), skin melanoma (C43), female breast (C50), cervix uteri (C53), corpus uteri (C54), ovary (C56), prostate (C61), testis (C62), kidney (C64-66), bladder (C67), brain and central nervous system (C70-72), thyroid (C73), Hodgkin’s disease (C81), Non-Hodgkin’s disease (C82-85, C96), multiple myeloma (C88, C90), leukaemia (C91-95), all cancer sites (except non-melanoma skin – C00-96) – and geographic region (North, Northeast, Midwest, Southwest and South).

The relative increase in premature deaths between the last observed (2011–2015) and the last predicted (2026–2030) 5-year period was calculated. Age standardised mortality rates (ASR) per 100,000 were calculated adjusted to the world population (16) and truncated to the 30-69 age group population.

#### Predictions

Predictions were performed using Nordpred software package for R (version 3.2.0) developed by the Cancer Registry of Norway (17). NORDPRED is widely used to make long-term predictions of cancer incidence and mortality. The model requires at least fifteen years of consecutive data (three five-year periods). For each combination of region, cancer topography, sex, and observed periods, predictions were calculated. Then, changes in the number of cases in the last projected period, 2026-2030, compared to the last observed period, 2011-2015, were calculated. This global change in the number of deaths is broken down into two components: one part due to the change in the risk of dying from cancer, and the other part due to changes in population size and age distribution (18, 19). These two components can be different from zero and present a positive or negative direction. The calculation of these components follows the equation:


Δtot=Δrisk+Δpop=(Nfff−Noff)+(Noff−Nooo)

Where Δtot is the total change, Δrisk is the change as a function of risk, Δpop is the change as a function of population, Nooo is the number of observed cases, Nfff is the number of projected cases, and Noff is the number of cases expected when mortality rates increase during the observed period.

### Probability of changes in cancer deaths

As a final step, in order to assess whether a one-third reduction in premature deaths (SDG target) is likely to be met, we converted the predicted changes into probabilities (20). The calculation of projected premature death probabilities for the 2011-2015 and 2026-2030 periods follows the methodology recommended by WHO (20) for the five-year age groups between 30 and 69 years, by topography, by sex, regions and Brazil, from registered deaths and population data.

Age-specific mortality rates are initially calculated for each age group using the formula:


$$5*M_{x\;}=\;\frac{Total\;cancer\;deaths\;between\;exact\;age\;x\;and\;exact\;age\;x+5}{Total\;population\;between\;exact\;age\;x\;and\;exact\;age\;x+5}$$

Then, the calculation of the probability of death in each 5-year age group, using the formula:


$$5*q_{x}=\;\frac{5*M_{x\;}*5}{1+\;5*M_{x\;}*2.5}$$

Finally, the probability of death between 30 and 69 years old, regardless of other causes of death, is given by:


$$40*q_{30}\;=\;1-\;\prod_{x=30}^{65}\left(1\;–\;5*q_{x}\right)$$

The probabilities of death by topography were also projected for the period from 2026 to 2030, with a reduction of 33% until 2030, considering the probabilities of the last observed period (2011–2015).

Finally, the relative differences in the probabilities of premature death between 2011-2015 and 2026-2030 were compared, in order to observe whether the objective of reducing the probabilities of premature death by 33% will be achieved. All analyses and graphs (with the exception of the predictions) were performed using Stata 14 (21).

## Results

Between 2001 and 2015, approximately 1.4 million premature deaths due to cancer occurred in Brazil (743,346 in males and 676,029 in females). The relative increase in premature deaths between the last observed (2011–2015) and the last predicted (2026–2030) 5-year period was 27% (n=75,341) in males and 35% (n=90,513) in females (**Table 2**) with the largest proportion attributable to population changes (population changes: 46% in males and 41% in females; risk factor changes -19% in males and -7% in females) (**Table 2**).

**Table 2:** Relative changes in number of deaths, total percentage of change and percentage attributed to specific changes in risk factors (Risk) and population (Pop), comparison between the last predicted (2026-2030) and the last observed period (2011-2015).

	Brazil				North				Northeast			
	Total change		Specific changes (%)		Total change		Specific changes (%)		Total change		Specific changes (%)	
	n	%	Risk	Pop	n	%	Risk	Pop	n	%	Risk	Pop
**Males**												
All Cancer Sites (C00-96)	75.341	27	-19	46	9.517	70	3	67	20.12	37	-8	45
Mouth and pharynx (C00-14)	4.726	19	-23	42	725	92	25	67	1.566	33	-10	44
Oesophagus (C15)	3.841	15	-29	44	503	83	15	68	2.698	62	17	45
Stomach (C16)	5.058	17	-29	46	1.366	53	-15	68	1.328	21	-24	45
Colorectum (C18-21)	13.925	58	13	45	1.168	150	85	65	2.859	90	46	44
Liver (C22)	6.756	37	-9	46	438	39	-28	67	1.316	32	-14	46
Pancreas (C25)	6.749	47	0	47	341	59	-11	70	1.685	68	22	46
Lung with trachea (C33-34)	3.037	7	-42	49	802	40	-31	71	1.51	20	-27	48
Prostate (C61)	4.794	28	-29	57	392	41	-36	77	870	19	-33	52
**Females**												
All Cancer Sites (C00-96)	90.513	35	-7	41	10.922	75	7	69	22.948	39	-5	43
Mouth and pharynx (C00-14)	932	22	-20	42	87	49	-25	75	224	20	-26	46
Oesophagus (C15)	551	10	-36	46	86	61	-16	78	397	33	-15	48
Stomach (C16)	4.337	29	-12	42	461	42	-30	72	1.176	32	-12	44
Colorectum (C18-21)	12.922	54	11	43	665	83	11	72	3.807	101	56	45
Liver (C22)	3.868	34	-11	45	134	21	-55	76	632	19	-28	47
Pancreas (C25)	6.423	55	8	47	75	18	-58	76	1.369	63	15	48
Lung with trachea (C33-34)	13.618	44	-2	46	626	49	-29	78	3.629	57	9	48
Female Breast (C50)	21.696	39	2	38	2.672	111	44	67	6.13	51	10	41
Cervix Uteri (C53)	6.727	23	-11	34	1.756	46	-16	63	1.682	19	-20	39
Ovary (C56)	3.998	34	-7	41	453	84	16	69	1.16	44	1	43
	Southeast				South				Midwest			
	Total change		Specific changes (%)		Total change		Specific changes (%)		Total change		Specific changes (%)	
	n	%	Risk	Pop	n	%	Risk	Pop	n	%	Risk	Pop
**Males**												
All Cancer Sites (C00-96)	28.921	21	-21	42	10.831	19	-21	40	9200	50	-13	63
Mouth and pharynx (C00-14)	1.166	9	-29	38	581	13	-21	34	842	52	-7	59
Oesophagus (C15)	674	5	-35	40	-28	0	-37	37	773	50	-12	63
Stomach (C16)	1.677	12	-31	43	676	13	-26	40	236	14	-50	64
Colorectum (C18-21)	6.297	47	4	42	2.837	57	17	39	1.473	90	29	61
Liver (C22)	2.657	32	-11	43	1.601	46	7	39	526	46	-17	63
Pancreas (C25)	2.712	38	-5	43	1.599	50	9	41	584	62	-4	66
Lung with trachea (C33-34)	537	3	-43	46	-314	-3	-47	44	1.097	38	-30	68
Prostate (C61)	1.987	26	-29	55	697	25	-30	55	675	55	-22	78
**Females**												
All Cancer Sites (C00-96)	34.807	28	-8	36	12.228	26	-9	35	9.188	53	-7	60
Mouth and pharynx (C00-14)	358	18	-18	36	95	15	-21	36	186	61	0	62
Oesophagus (C15)	39	2	-37	39	-51	-4	-45	41	104	30	-38	68
Stomach (C16)	1.424	21	-16	37	612	26	-9	35	621	67	7	60
Colorectum (C18-21)	6.138	46	9	37	1.623	37	0	37	992	62	0	62
Liver (C22)	1.813	37	-3	40	1.159	61	21	40	205	32	-34	66
Pancreas (C25)	3.349	56	14	42	1.001	42	0	42	540	78	9	69
Lung with trachea (C33-34)	4.464	31	-9	40	3.209	47	8	39	1.68	86	17	69
Female Breast (C50)	7.88	29	-3	32	3.063	33	3	30	2.664	62	6	56
Cervix Uteri (C53)	1.673	17	-12	29	909	22	-3	25	473	22	-28	49
Ovary (C56)	1.225	22	-14	35	399	20	-15	35	674	81	21	60

**Figure 2:**
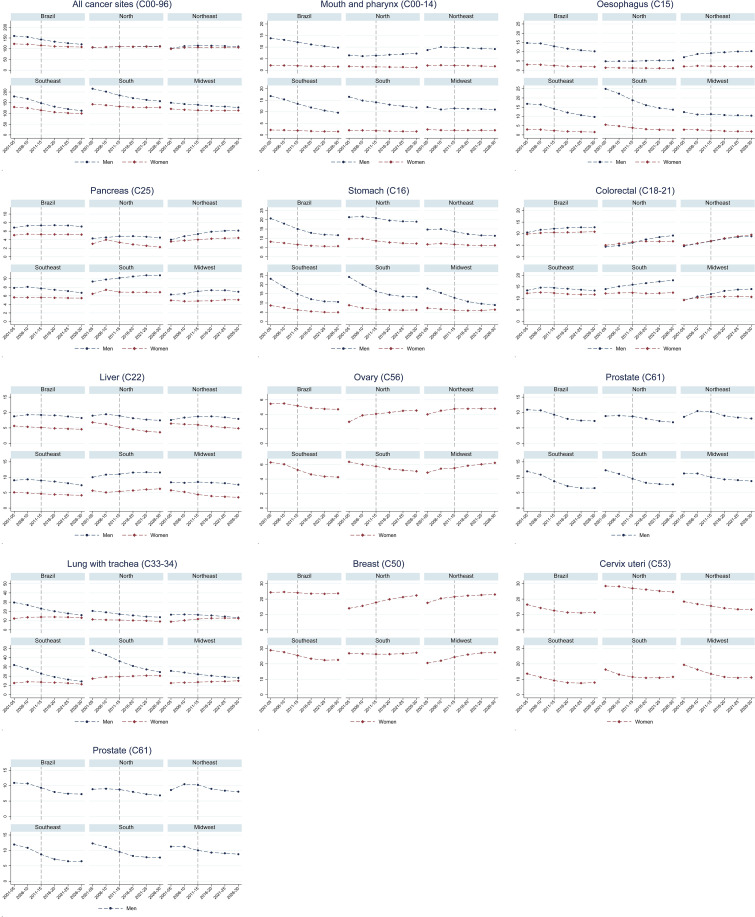
Observed and predicted age-standardised mortality rates (ASR) for people aged 30-69 years, by sex, region and period, 2001-2030. All cancer sites, excluding skin non-melanoma.

The predictions show that the ASR (per 100,000) for all cancers is likely to decrease in Brazil, among both males (from 145.8 in 2011-2015 to 127.1 in 2026-2030; -14.8%) and females (118.3 in 2011-2015 to 112.9 in 2026-2030; -4.7). This was observed in all regions except the North, where a slight increase is predicted (1.3% in males and 3.5% in females; **Table 3** and **Figure 2**).

**Table 3:** Observed and predicted number of deaths and age-standardised mortality rates (ASR) for people aged 30-69 years, by sex, region and calendar period, 2001-2030.

	Observed						Predicted						ASR % change
	2001-2005		2006-2010		2011-2015		2016-2020		2021-2025		2026-2030		2011-2015
	n	ASR	n	ASR	n	ASR	n	ASR	n	ASR	n	ASR	2026-2030
Men													
Brazil	216,241	159.2	247,141	154.3	279,964	145.8	306,552	138.3	332,706	132.3	355,305	127.1	-12.9
North	8,725	106.9	10,580	107.2	13,590	109.6	16,534	111.1	19,774	111.4	23,107	111.0	1.3
Northeast	34,755	101.5	44,384	112.1	53,701	114.2	60,226	113.6	67,073	111.6	73,821	108.5	-5.0
Southeast	111,936	179.3	123,987	168.4	136,563	155.6	147,667	145.6	157,863	138.2	165,484	132.7	-14.7
South	47,927	215.4	53,133	201.8	57,816	184.2	62,022	171.7	65,762	163.0	68,647	157.0	-14.8
Midwest	12,897	143.8	15,057	138.1	18,295	134.8	21,277	130.9	24,504	128.0	27,495	124.3	-7.8
Females													
Brazil	192,118	122.4	222,935	120.8	260,976	118.3	292,832	115.6	323,423	113.8	351,489	112.9	-4.5
North	8,969	105.4	11,283	108.0	14,475	110.2	17,999	113.1	21,646	113.8	25,397	114.2	3.6
Northeast	40,151	99.2	49,259	105.2	59,251	106.8	67,096	106.2	74,633	104.7	82,199	103.2	-3.3
Southeast	95,141	130.0	107,220	124.9	123,430	120.8	136,450	117.0	148,442	115.0	158,237	114.3	-5.4
South	36,103	142.8	41,060	138.6	46,585	132.6	51,229	128.0	55,467	125.6	58,813	124.4	-6.2
Midwest	11,754	121.9	14,115	117.2	17,240	114.0	20,208	111.2	23,332	109.7	26,428	109.3	-4.2

**Figure 2** show percentage changes and premature mortality trends for specific sites. A decrease in premature ASRs for mouth and pharynx, and oesophageal cancer is expected among males in Brazil as a whole, and in the South and Southeast, but not among females. Premature mortality percentage changes due to demographic changes are higher in the Midwest and in the North compared to the other regions. Stomach cancer premature mortality rates are projected to decrease among men nationally and in all regions, but remain stable in the Northeast and the Midwest among females. For prostate and cervical cancer, decreases in premature mortality are projected in all regions. Colorectal cancer premature mortality rates are expected to increase in men nationally and all regions, except in the Southeast (where rates are expected to remain stable); among females, increasing rates are expected only in the North and Northeast. For lung cancer, the results show a reduction in males and a slight increase in females, with increasing trends in the last predicted periods. Breast cancer mortality results show an increase in rates in the North, Northeast and Midwest, a reduction in the South and stability in the Southeast (**Figure 2**).

**Figure 3:**
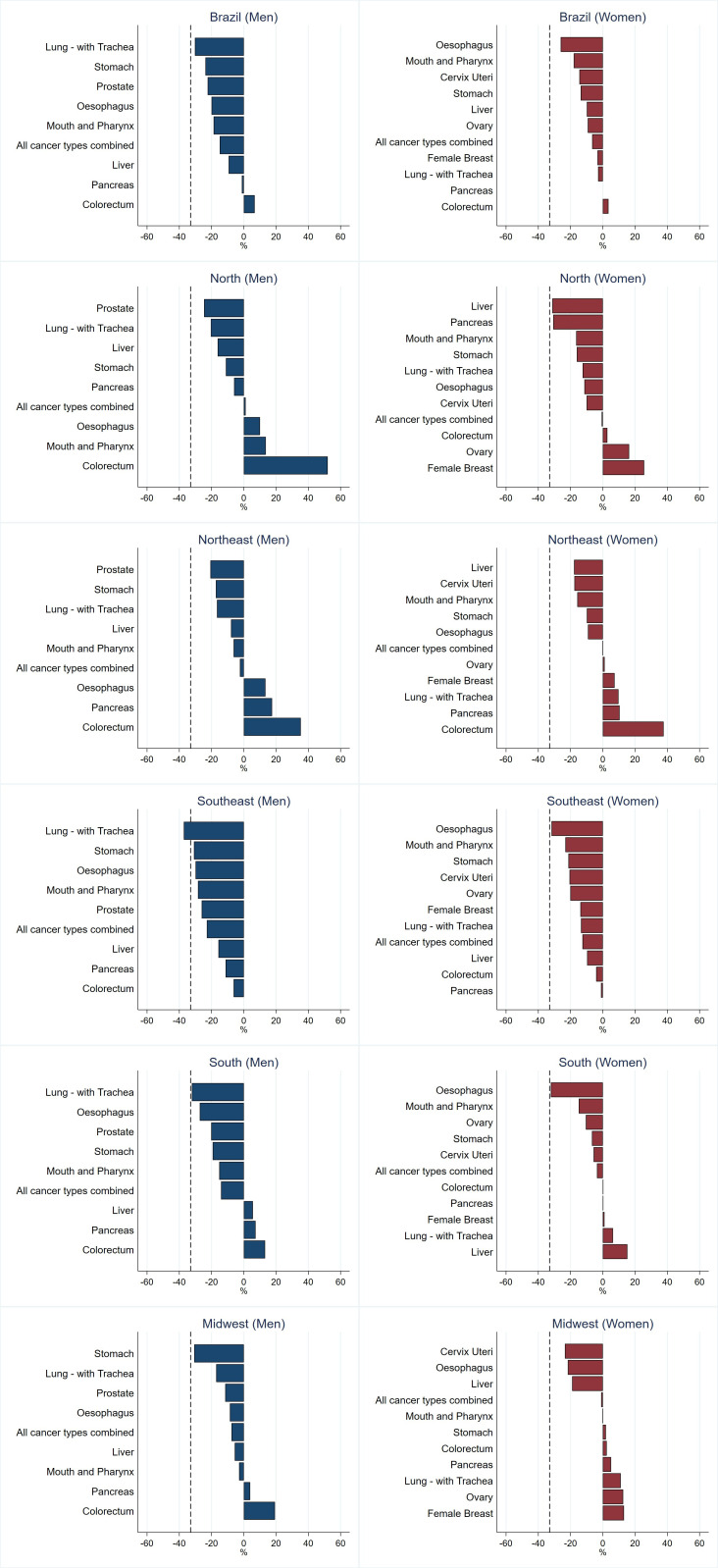
Probability of death in the last predicted period (2026–2030) compared to the last observed period (2011-2015) in relation to the 33% reduction target 3.4 (shown as a dashed black line).

**Figure 3** shows the difference between the observed (2011–2015) and predicted (2026–2030) premature mortality probabilities, in relation to the SDG target of 33% decrease. The results show that this objective will not be achieved in Brazil or the regions for most cancer sites, except for lung (Southeast, males) and oesophageal (South, females) cancers. The Southeast is the only region where all the selected tumours shown in **Figure 3** are likely to decrease among men and women.

## Discussion

This study shows that the UN SDG target of reducing premature cancer mortality by one-third in Brazil by 2030 is not expected to be met, regarding cancer. We observed marked regional disparities in premature mortality ASRs where decreases were mainly evident in the South and Southeast region, the most affluent regions of Brazil, while the North and Northeast – less affluent - showed a mix of patterns ultimately resulting in a minimal reduction of expected future premature mortality from cancer. The Southeast is likely to be the only region to achieve the UN SDG target, and this was only seen for lung cancer in males. The most favourable predictions in males are expected for lung, stomach, liver and prostate cancers and in females for cancers of the mouth and pharynx, oesophagus, liver and cervix, but even for these cancers, the projected decreases are far below the one-third target. Colorectal cancer stands out, with mortality expected to increase in all regions in both males and females, with the exception of the Southeast where stability is expected.

Although the age-standardized mortality rates are projected to decline slightly nationally in both sexes (by 15% in males and 5% in females), the number of deaths is projected to increase. Changes in the number of deaths are important to plan health services and facilities, while assessment of mortality rates is valuable for the evaluation of cancer control programs.

To explain the results of premature mortality predictions and their relationship to targets as set by the UN SDGs, we need to consider that changes in the risk of death from a particular type of cancer can arise from three factors: changes in exposure to cancer risk factors (primary prevention), earlier detection and cancer diagnosis (secondary prevention), and adequate management of the disease (tertiary prevention).

Although Brazil is not predicted to reach the UN SDG premature mortality reduction target by 2030, this study does reveal where significant reductions have, and will arise. For example, in the case of primary prevention, it is noteworthy that premature mortality rates for several of the cancers for which tobacco exposure is a causal factor are projected to decrease (albeit not by one third) primarily due to primary prevention. Brazil has been successful in reducing smoking prevalence through several public policies (22), such as price increases, tobacco-free area laws, marketing restrictions, warnings, and mass media smoking cessation campaigns and access to pharmacological treatment in public health services. Smoking prevalence has declined from 32.4% in 1989 to 18.7% in 2013 (22, 23). Given the lag time for the development of cancer following tobacco exposure, this is likely to explain the expected reduction in the tobacco related cancers.

### Primary prevention

In terms of population attributable fractions, infections are the second main potentially modifiable risk factor for cancer in many settings, mainly driven by HPV infection (24). Cervical cancer is highly preventable through both primary (HPV vaccination) and secondary (screening with Pap smears or HPV testing) prevention. Pap smears are freely available in the Brazilian public health system, but are offered opportunistically rather than within a systematic organized call and recall-based programme, and uptake is lower in the poorest regions of the country and among those with a lower education level (25). The tetravalent HPV vaccine was introduced in Brazil in 2014, but coverage is still low, 58.4% of eligible girls are fully vaccinated (26). Efforts to improve vaccination coverage rates could have a significant impact on reducing the burden of disease in the coming years. Pap smear tests, HPV testing and HPV vaccination are cost-effective measures to avoid cervical cancer (27) and need to be reinforced in the regions with highest mortality rates (North and Northeast), combined with enhanced access to public health system for treatment of pre-cancerous lesions. In terms of other infections, helicobacter pylori infection is responsible for 57% of stomach cancers in Brazil; hepatitis B virus (HBV) and hepatitis C virus (HCV) infections are estimated to be responsible for 7% and 15% of liver tumours, respectively (24). The health system offers HBV vaccination free at the primary health centres, and screening and treatment for HCV, but our results suggest that increases in the coverage are necessary.

Obesity is an established potentially modifiable risk factor for oesophageal (adenocarcinoma), colorectal, pancreas, and breast (post-menopausal) cancers (28), which could (in theory) be highly influenced through public health policies aiming to increase physical activity and healthy eating. A possible explanation for the projected increase in premature mortality rates from colorectal cancer, and lack of a notable decrease in breast cancer rates, is the increase in obesity observed in Brazil, which follows a global trend (28). Obesity prevalence increased from 2002 to 2013, in all Brazilian regions, more markedly in females than in males (29). Promoting a healthy diet and physical activity has the potential to revert obesity and protect against several cancers. Rather than only relying on individuals changing their behaviours, intersectoral public policies are necessary to promote healthy environments, as was successfully done with tobacco control.

Alcohol consumption is recognized as a risk factor for colorectal, oral cavity, oesophageal, stomach, pancreatic, liver, larynx and postmenopausal breast cancer (24). Brazil does not have effective public policies to control alcohol consumption. The regions North, Northeast and Midwest have the highest prevalence of heavy drinking (23.1%, 25.5% and 24.0%) (30). The predictions in these regions show increase or stability in premature mortality rates for the majority of these tumours, the highest increases observed for colorectal and breast cancers. Adopting and enforcing measures to control harmful use of alcohol are cost-effective (27) ways to avoid new cancer cases, and consequently cancer deaths in the future.

#### Secondary prevention

In terms of secondary prevention, there is considerable potential for this for colorectal cancer, and a variety of screening tests have been shown to be efficacious and cost-effective (31). In Brazil, although colorectal cancer is the third most incident cancer (32), there is no organised early detection strategy or screening programme at public system level; given our findings, there is an urgent need for this.

Although mammographic screening is available for breast cancer, diagnosis is performed opportunistically, according to national guidelines, but regional differences are present in the distribution of diagnostic and treatment services, and consequently in access. Overall 51.0% of eligible females underwent mammographic screening on the public health system, with further 10% screened privately (33). However, this varied by region; the lowest prevalence was observed in the North (38.7%) and the highest in the Southeast (67.9%). Given the increasing predicted trends in breast cancer premature mortality, improving access to breast cancer screening and treatment may be effective in reducing the burden of breast cancer.

### Tertiary prevention

In terms of tertiary care, timely access to effective treatment is fundamental to improve cancer outcomes in the middle-term. Treatment delays – need to be identified and corrected by local health authorities and decision-makers. A recent study analysed the network of cancer accredited services, and the more deprived regions are those in more need of accredited services (34). At the time of the study, Brazil had 299 public high-complexity oncology services in 173 of its 5570 cities. Access to secondary and tertiary care for people living in distant areas needs to be better coordinated and provided by the public health system (34). Probably part of the decrease in mortality of certain cancers in the Southeast is associated with the greater availability and access to cancer accredited hospitals and services. Regarding equity, it is also critical to provide adequate access to palliative services and end-of-life support.

In terms of limitations of the study, it is important to remember that these data are based on the cancers that resulted from exposures to risk factors in the past (and sometimes many years in the past); these exposures will not necessarily continue in the future. Detailed regional data (and, in many instances, national) data on the prevalence of risk factors is not available. It is also challenging to compare our results with those from other studies because usually all age groups are included, while we limited consideration to deaths that would be judged premature according to the UN definition. This analysis was undertaken before the COVID-19 pandemic and did not take probable changes in mortality into account. This is preferable than including the years of the pandemic, due to mortality patterns that probably will continue.

The economic burden of NCDs at the household level needs to be considered in order to alleviate global poverty. For this reason, political commitments to effective and equitable national surveillance for major cancers are needed to reduce the burden of premature deaths in the country. It is necessary to prioritise social policies and to adequately plan primary prevention, early detection and disease management according to regional differences to overcome those major disparities in premature cancer-related premature mortality. In conclusion, this analysis shows that, for cancer in general, and several of the major cancer sites, the UN SDG 3.4 will not be met in Brazil as a whole or in its regions. Action need to be taken now, but the effects will be seen in the coming decades and not by 2030, due to the natural history of disease.

## Data availability statement

Publicly available datasets were analyzed in this study. This data can be found here: https://datasus.saude.gov.br/mortalidade-desde-1996-pela-cid-10.

## Author contributions

MC: Conceptualization, data curation, funding acquisition, methodology, project administration, analysis, supervision, validation, writing, review and editing DB: data curation, methodology, analysis, validation, writing, review and editing LL: data curation, methodology, analysis, validation, writing, review and editing LB: data curation, methodology, analysis, validation, writing, review and editing AS: data curation, validation, review and editing PH: methodology, validation, writing, review and editing LS: Conceptualization, methodology, validation, writing, review and editing AP: Conceptualization, methodology, validation, writing, review and editing IS: Conceptualization, methodology, validation, writing, review and editing. All authors contributed to the article and approved the submitted version.

## References

[1] NCD Countdown 2030 collaborators. NCD Countdown 2030: worldwide trends in non-communicable disease mortality and progress towards Sustainable Development Goal target 3.4. Lancet (2018) 392(10152):1072–88. doi: 10.1016/S0140-6736(18)31992-5 30264707

[2] Murray CJL Barber RM Foreman KJ Ozgoren AA Abd-Allah F Abera SF. Global, regional, and national disability-adjusted life years (DALYs) for 306 diseases and injuries and healthy life expectancy (HALE) for 188 countries, 1990-2013: Quantifying the epidemiological transition. Lancet (2015) 386(10009):2145–91. doi: 10.1016/S0140-6736(15)61340-X PMC4673910 26321261

[3] World Health Organization. From Burden to “Best Buys”: Reducing the Economic Impact of Non-Communicable Diseases in Low- and Middle-Income Countries. (2011) Geneva: WHO.

[4] Cao B Bray F Ilbawi A Soerjomataram I. Effect on longevity of one-third reduction in premature mortality from non-communicable diseases by 2030: a global analysis of the sustainable development goal health target. Lancet Global Health (2018) 6(12):e1288–96. doi: 10.1016/S2214-109X(18)30411-X 30420032

[5] United Nations General Assembly. Transforming our world: the 2030 agenda for sustainable development. (2015) 16301:1–35.

[6] Wild CP. The role of cancer research in noncommunicable disease control. JNCI J Natl Cancer Institute. (2012) 104(14):1051–8. doi: 10.1093/jnci/djs262 PMC3402142 22781435

[7] ANS (Agência Nacional de Saúde Suplementar). Dados e indicadores do setor (2020). Available at: http://www.ans.gov.br/perfil-do-setor/dados-e-indicadores-do-setor.

[8] de Albuquerque MV d’Ávila Viana AL de Lima LD Ferreira MP Fusaro ER Iozzi FL. Regional health inequalities: changes observed in Brazil from 2000-2016. Ciênc saúde coletiva. (2017) 22:1055–64. doi: 10.1590/1413-81232017224.26862016 28444033

[9] Giovanella L Mendoza-Ruiz A Pilar A de CA Rosa MCD Martins GB Santos IS. Universal health system and universal health coverage: assumptions and strategies. Cien Saude Colet. (2018) 23(6):1763–76. doi: 10.1590/1413-81232018236.05562018 29972485

[10] Brasil, Ministério da Saúde, DATASUS. Sistema de informações sobre mortalidade (2022). Available at: https://datasus.saude.gov.br/transferencia-de-arquivos/.

[11] IBGE. Portal do IBGE. Rio de Janeiro, Brazil: IBGE (2020). Available at: https://www.ibge.gov.br/.

[12] United Nations Statistics Division. Coverage of birth and death registration (2016). Available at: https://unstats.un.org/unsd/demographic-social/crvs/.

[13] Silva GA Gamarra CJ Girianelli VR Valente JG. Cancer mortality trends in Brazilian state capitals and other municipalities between 1980 and 2006. Rev Saude Publica. (2011) 45(6):1009–18. doi: 10.1590/S0034-89102011005000076 22127651

[14] Loos AH Bray F McCarron P Weiderpass E Hakama M Parkin DM. Sheep and goats: separating cervix and corpus uteri from imprecisely coded uterine cancer deaths, for studies of geographical and temporal variations in mortality. Eur J Cancer. (2004) 40(18):2794–803. doi: 10.1016/j.ejca.2004.09.007 15571963

[15] de Souza Giusti ACB de Oliveira Salvador PTC Dos Santos J Meira KC Camacho AR Guimarães RM. Trends and predictions for gastric cancer mortality in Brazil. World J Gastroenterol (2016) 22(28):6527–38. doi: 10.3748/wjg.v22.i28.6527 PMC4968132 27605887

[16] Doll R Payne P Waterhouse JAH. eds. Cancer Incidence in Five Continents. Geneva: Union Internationale Contre le Cancer (1996) vol. I. Available at: https://publications.iarc.fr/Non-Series-Publications/Other-Non-Series-Publications/Cancer-Incidence-In-Five-Continents-Volume-I-1966.

[17] Fekjaer H Bjorn M. Nordpred: Fit power5 and poisson age-Period-Cohort models to calculate prediction of cancer incidence and mortality (2022). Available at: https://rdrr.io/github/haraldwf/nordpred/man/nordpred.html.

[18] Møller B Fekjaer H Hakulinen T Sigvaldason H Storm HH Talbäck M. Prediction of cancer incidence in the Nordic countries: empirical comparison of different approaches: COMPARISON OF METHODS FOR INCIDENCE PREDICTION. Statist Med (2003) 22(17):2751–66. doi: 10.1002/sim.1481 12939784

[19] Jerez-Roig J Souza DLB Medeiros PFM Barbosa IR Curado MP Costa ICC. Future burden of prostate cancer mortality in Brazil: a population-based study. Cad Saude Publica. (2014) 30(11):2451–8. doi: 10.1590/0102-311X00007314 25493998

[20] World Health Organization. Noncommunicable diseases global monitoring framework. Geneva, Switzerland: World Health Organization (2013). Available at: https://www.who.int/publications-detail-redirect/ncd-surveillance-global-monitoring-framework. Accessed 13/12/2022

[21] StataCorp. Statistical software for data science. Texas: College Station (2015) 14. Available at: https://www.stata.com/.

[22] Szklo AS de Almeida LM Figueiredo VC Autran M Malta D Caixeta R. A snapshot of the striking decrease in cigarette smoking prevalence in Brazil between 1989 and 2008. Prev Med fevereiro (2012) 54(2):162–7. doi: 10.1016/j.ypmed.2011.12.005 22182479

[23] Szklo AS de Souza MC Szklo M de Almeida LM. Smokers in Brazil: who are they? Tob Control. (2016) 25(5):564–70. doi: 10.1136/tobaccocontrol-2015-052324 26292700

[24] e Silva GA de Moura L Curado MP Gomes F da S Otero U de Rezende LFM. The fraction of cancer attributable to ways of life, infections, occupation, and environmental agents in Brazil in 2020. PloS One (2016) 11(2):e0148761. doi: 10.1371/journal.pone.0148761 26863517 PMC4749327

[25] de Oliveira MM de A Andrade SSC de Oliveira PPV e Silva GA da Silva MMA Malta DC. Pap-test coverage in women aged 25 to 64 years old, according to the national health survey and the surveillance system for risk and protective factors for chronic diseases by telephone survey, 2013. Rev Bras Epidemiologia (2018) 21, 1–10. doi: 10.1590/1980-549720180014 30156661

[26] Mendes Lobão W Duarte FG Burns JD de Souza Teles Santos CA Chagas de Almeida MC Reingold A. Low coverage of HPV vaccination in the national immunization programme in Brazil: Parental vaccine refusal or barriers in health-service based vaccine delivery? PloS One (2018) 13(11):e0206726. Angelillo IF, organizador. doi: 10.1371/journal.pone.0206726 30418980 PMC6231618

[27] World Health Organization. Global action plan for the prevention and control of NCDs 2013-2020. Geneva, Switzerland: World Health Organization (2020). Available at: https://www.who.int/publications-detail-redirect/9789241506236.

[28] World Cancer Research Fund International. Diet, nutrition, physical activity and cancer: a global perspective: a summary of the third expert report. London: World Cancer Research Fund International (2018). p. 112.

[29] Gomes DCK Sichieri R Junior EV Boccolini CS de Moura Souza A Cunha DB. Trends in obesity prevalence among Brazilian adults from 2002 to 2013 by educational level. BMC Public Health (2019) 19(1):965. doi: 10.1186/s12889-019-7289-9 31319818 PMC6637516

[30] Garcia LP de Freitas LRS. Consumo abusivo de álcool no brasil: resultados da pesquisa nacional de saúde 2013. Epidemiol Serv Saúde. (2015) 24:227–37. doi: 10.5123/S1679-49742015000200005

[31] Lauby-Secretan B Vilahur N Bianchini F Guha N Straif K. International agency for research on cancer handbook working group. the IARC perspective on colorectal cancer screening. N Engl J Med (2018) 378(18):1734–40. doi: 10.1056/NEJMsr1714643 PMC6709879 29580179

[32] Instituto Nacional de Câncer. Estimativa 2020: incidência de câncer no brasil. Rio de Janeiro, Brazil: Instituto Nacional de Câncer (2020). Available at: https://www.inca.gov.br/publicacoes/livros/estimativa-2020-incidencia-de-cancer-no-brasil.

[33] e Silva GA de Souza-Júnior PRB Damacena GN Szwarcwald CL e Silva GA de Souza-Júnior PRB. Detecção precoce do câncer de mama no brasil: dados da pesquisa nacional de saúde, 2013 Vol. 51. Revista de Saúde Pública (2017). Available at: http://old.scielo.br/scielo.php?script=sci_abstract&pid=S0034-89102017000200303&lng=en&nrm=iso&tlng=pt. 10.1590/S1518-8787.2017051000191 PMC5676402 28591356

[34] da Silva MJS O’Dwyer G Osorio-de-Castro CGS. Cancer care in Brazil: structure and geographical distribution. BMC Cancer. (2019) 19(1):987. doi: 10.1186/s12885-019-6190-3 31647005 PMC6806503

